# Value of Contrast Transesophageal Echocardiography in the Detection of Intrapulmonary Vascular Dilatations in Hepatosplenic Schistosomiasis

**DOI:** 10.5935/abc.20190200

**Published:** 2019-11

**Authors:** Aparecida de Gouvea, Claudio Henrique Fischer, Jaquelina Sonoe Ota Arakaki, Frederico José Mancuso, Paulo Brant, Valdir Ambrósio Moisés, Orlando Campos Filho

**Affiliations:** 1 Universidade Federal de São Paulo - Escola Paulista de Medicina, São Paulo, SP - Brazil

**Keywords:** Echocardiography, Transesophageal/methods, Dilatation, Pathologic, Schistosomiasis, Hypoxia, Cardiac Output, Liver Injury, Chronic/physiopathology

## Abstract

**Background:**

Hepatopulmonary syndrome (HPS), found in cirrhotic patients, has been little studied in hepatosplenic schistosomiasis (HSS) and includes the occurrence of intrapulmonary vascular dilatations (IPVD). Contrast transesophageal echocardiography (cTEE) with microbubbles is more sensitive than contrast transthoracic echocardiography (cTTE) with microbubbles in the detection of IPVD in cirrhosis.

**Objective:**

To assess the performance of the cTEE, compared with that of cTTE, in detecting IPVD for the diagnosis of HPS in patients with HSS.

**Methods:**

cTEE and cTTE for investigation of IPVD and laboratory tests were performed in 22 patients with HSS. Agitated saline solution was injected in peripheral vein during the cTEE and cTTE procedures. Late appearance of the microbubbles in the left chambers indicated the presence of IPVD. Results of the two methods were compared by the Student’s t-test and the chi-square test (p < 0.05).

**Results:**

cTEE was performed in all patients without complications. Three patients were excluded due to the presence of patent foramen ovale (PFO). The presence of IPVD was confirmed in 13 (68%) of 19 patients according to the cTEE and in only six (32%, p < 0.01) according to the cTTE. No significant differences in clinical or laboratory data were found between the groups with and without IPVD, including the alveolar-arterial gradient. The diagnosis of HPS (presence of IPVD with changes in the arterial blood gas analysis) was made in five patients by the cTEE and in only one by the cTTE (p = 0.09).

**Conclusion:**

In HSS patients, cTEE was safe and superior to cTTE in detecting IPVD and allowed the exclusion of PFO.

## Introduction

Intrapulmonary vascular dilatations (IPVD) are the anatomical substrate for clinical changes, particularly hypoxemia, observed in patients with chronic liver disease, portal hypertension or congenital or acquired portosystemic shunt, and constitute the hepatopulmonary syndrome (HPS) triad.^1-3^ However, IPVD and HPS have been little described in patients with hepatosplenic schistosomiasis (HSS) and portal hypertension, even in those with normal liver function.^4,5^

Diversion of venous blood through the liver, observed in portal hypertension in cirrhotic or HSS patients, or even in cavopulmonary anastomosis (Glenn procedure), is the pathophysiological determinant for IPVD. The imbalance between vasoconstrictor and vasodilator substances, dependent on hepatic synthesis or metabolization, favor the accumulation of vasodilator agents in the lungs, including the nitric oxide which plays an important role in this mechanism.^6,7^ Dilated capillaries, combined with increased cardiac output, found in hepatic dysfunction without cardiac or lung disease, lead to a ventilation perfusion mismatch, resulting in hypoxemia or alveolar-arterial gradient (A-aO_2_).

IPVD can be identified by microbubble contrast transthoracic echocardiography (cTTE). Although it has been considered the standard diagnostic method,^8^ the method has limitations including inadequate window or false positive results for the diagnosis of IPVD, due to the presence of left-to-right shunt through the patent foramen ovale (PFO).^9^ Contrast transesophageal echocardiography (cTEE) with microbubbles provides better image definition and higher diagnostic accuracy than cTTE for identification and classification of IPVD in cirrhotics.^10-12^ In addition, cTEE enables a precise, direct detection of PFO since it enables the early detection of microbubbles in the left atrium.

Although some studies have reported the use of cTTE in patients with HSS,^4^ there is no study on the use of cTEE in these patients. Considering the high diagnostic performance of cTEE for the detection of IPVD in cirrhotics, the present study aimed to establish the role of cTEE in identifying and quantifying IPVD in HSS and compare with the results obtained by cTTE.

## Methods

### Patients

Twenty-nine patients with diagnosis of HSS without pulmonary hypertension were first evaluated for the presence of HPS, including the detection of IPVD by cTTE. All patients met epidemiological, clinical (hepatosplenomegaly), laboratorial (Schistosomiasis mansoni eggs in feces or rectal biopsy) and ultrasonographic criteria (periportal fibrosis) for HSS. Liver function was relatively preserved in all patients, and only one patient had ascites at clinical examination. Seven patients were excluded - two for previous splenectomy, two for pulmonary disease, one for hepatic cirrhosis, one for portal vein thrombosis and one for poor echocardiographic image quality. After the cTEE, another three patients were excluded for PFO, and the final study group was composed of 19 patients (HSS group). Seventeen (89%) patients had esophageal varices at endoscopy and previous sclerotherapy. No patient had left ventricular dysfunction, heart valve disease, suspected or proven coronary artery disease, or congenital heart disease. Demographic and clinical characteristic of HSS patients are described in Table 1.

**Table 1:** Demographic and clinical characteristics of patients with hepatosplenic schistosomiasis (HSS)

Age (years)	51.8 ± 8.4	(41-66)
Sex	10 men	9 women
Body surface (m^2^)	1.72 ± 0.14	(1.56 - 2.07)
Heart rate (bpm)	73 ± 16	(51-106)
SBP (mmHg)	139 ± 20	(105-178)
DBP (mmHg)	83 ± 15	(60-113)
Hemoglobin (g/dl)	13.7 ± 2.4	(8.9-18.8)
Hematocrit (%)	41.1 ± 5.3	(30.6-48.1)
Platelets (mil/mm^3^)	94.843 ± 60.391	(25.000-281.000)
Albumin (g/dL)	4.43 ± 0.40	(3.9-5.3)
AST (U/L)	34.8 ± 10.1	(24-59)
ALT (U/L)	31.5 ± 13.5	(17-77)
Alkaline phosphatase (U/L)	94 ± 26	(67-161)
INR	1.2 ± 0.18	(1.01-1.5)
PaO_2_ (mmHg)	80.2 ± 9.1	(60-97)
PaCO_2_ (mmHg)	36.7 ± 4.7	(28-45)
SatO_2_ (%)	95.8 ± 1.59	(94-98)
A-aO_2_ (mmHg)	10.9 ± 7.5	(2.63-26.68)

Values expressed as mean and standard deviation (variation range in parentheses). SBP: systolic blood pressure; DBP: diastolic blood pressure; AST: aspartate transaminase (U/L); ALT: alanine transaminase (U/L); INR: international normalized ratio; PaO_2_: partial oxygen pressure; PaCO_2_: partial pressure of carbon dioxide; SatO_2_: blood oxygen saturation; A-aO_2_: alveolar-arterial gradient.

Nineteen patients who underwent cTEE for detection of embolic events were selected as control of enhancement degree of the microbubble contrast agent. Control subjects were matched for age and sex with the HSS group. No patient of the HSS group had hepatic, cardiac or pulmonary disease, or PFO at cTEE.

All individuals of the HSS group and the control group had stable sinus rhythm, with normal left ventricular ejection fraction (>0.55), tricuspid regurgitation velocity (TRV) slower than 2.8 m/s^13^ and absence of indirect signs of pulmonary hypertension. All subjects signed the informed consent form and the study protocol was approved by the local ethics committee.

### Clinical evaluation

Laboratory tests were performed on blood samples from the HSS group patients for assessment of liver function, arterial blood gas analysis (sitting position, at rest and in environmental air), abdominal ultrasound, upper digestive endoscopy, chest radiography and spirometry were performed within three weeks after the cTTE. A-aO_2_ was calculated using the formula:

$$A-{\mathrm{aO}}_{2}=\left[{\mathrm{FiO}}_{2}\times \left(\mathrm{atm}-P\right)-{\mathrm{PACO}}_{2}/R\right]-{\mathrm{PaO}}_{2,}$$

where FiO_2_ is the fraction of inspired oxygen; atm is the atmospheric pressure in São Paulo city (690 mmHg); P is the vapour pressure of water at 37°C = 47mmHg; PACO_2_ is the alveolar pressure of carbon dioxide, assumed to be equivalent to the PaCO_2_; R is the respiratory exchange rate (V’CO_2_/V’O_2_), considered to be 0.8. Oxygen saturation was also measured (SatO_2_).

The diagnosis of HPS was defined as A-aO_2_ greater than 15 mmHg and/or PaO_2_ lower than 80 mmHg,^14^ combined with the presence of IPVD detected by microbubble cTTE.

### Echocardiography

All participants of the HSS and the control groups underwent cTTE followed by cTEE, using an HDI 7 device with 4 MHz transducer (Phillips Health Care) and a 3-7 MHz multiplane esophageal transducer, respectively.

**Figure 1:**
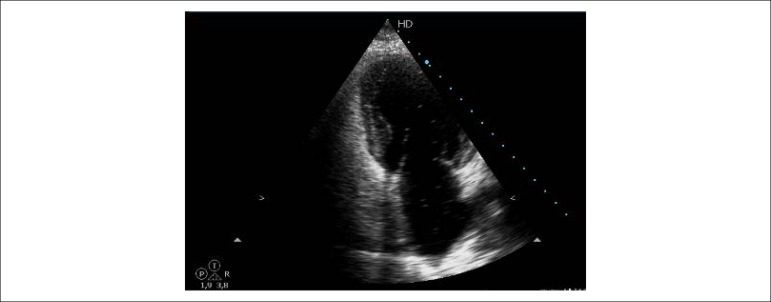
Apical four-chamber view of contrast transthoracic echocardiography. Opacification of the right chambers after four cardiac cycles (positive test for intrapulmonary vascular dilatations).^1^

cTTE was used for structural and functional evaluation of the heart, following the American Society of Echocardiography (ASE) recommendations.^15-17^ Left ventricular ejection fraction was calculated using the Simpson’s biplane method. Pulmonary arterial pressure was estimated from the tricuspid regurgitation. A contrast-enhanced test was performed by injection of saline after its manual agitation with air to create microbubbles. In the presence of IPVD, saline microbubbles appeared in the left chambers 4-6 heartbeats after their appearance in the right ventricle. An early appearance (< 3 heartbeats) was associated with intracardiac shunt, generally due to PFO, and the diagnosis was optimized by the Valsalva maneuver. The effect of the contrast agent was observed in the apical four chamber view and classified as present (positive contrast echocardiogram) or absent (negative contrast echocardiogram), without graduating the intensity (Figure 1). None of the control subjects showed a positive contrast test at cTTE.

In the cTTE, midazolam and fentanyl were used for sedation with continuous monitoring of SatO_2_, heart rhythm, and systemic blood pressure during the procedure, following well-established methods.^18^ Valsalva maneuver was performed whenever possible.

**Figure 2:**
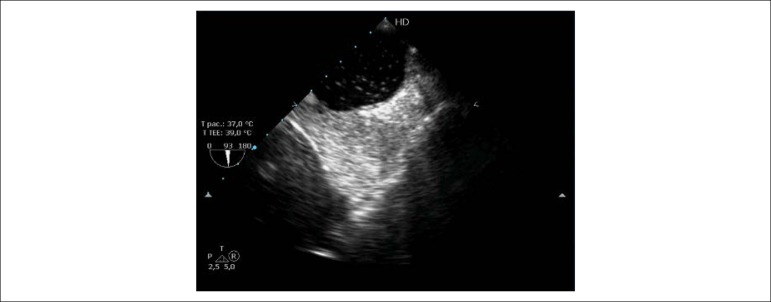
Bicaval view of contrast transesophageal echocardiography. Opacification of the right atrium after injection of saline contrast, with small, scattered microbubbles (mild contrast test) in the left atrium after four cardiac cycles

Esophageal varices were not considered contraindications to cTTE, except in the presence of active or evidence of recent bleeding (<15 days). After injection of agitated saline, contrast in the left atrium was quantified according to the classification proposed by Vedrinne et al.^10^ and modified by us: grade 0: no microbubbles; grade I (minimal) = occasional and punctiform microbubbles; grade II (mild) = the microbubble signal was unclear and diffuse, with incomplete opacification of the left atrium; grade III (moderate) = many microbubbles with homogeneous opacification of the left atrium, but with lower intensity than the right atrium; and grade IV (significant) = homogeneous distribution of the microbubbles between the two atria (Figure 2).

### Statistical analysis

Continuous variables were described as mean and standard deviation, or median and interquartile ranges. Categorical variables were described as proportions. The Student’s t-test was used for comparison of continuous variables between the groups, and the chi-square test, the Pearson’s test and the Fisher’s exact test used to compare the frequencies of categorical variables when necessary. A p < 0.05 was set for statistical significance.

## Results

Patients with HSS showed normal parameters of cardiac structure (chamber size, left ventricular mass and thickness), preserved systolic and diastolic functions (ejection fraction of 0.67 ± 0.04) and pulmonary systolic pressure of 27 ± 3 mmHg (TRV= 2.34 ± 0.14 m/s) at cTTE, which was performed without complications in all HSS patients. There was no case of hypoxia, arrythmia, neurological complications or upper gastrointestinal bleeding.

In the control group, 12 (65%) individuals showed grade I in the contrast test, and the other seven showed grade 0. Therefore, grade I was considered the normal response to the CTEE, and only contrast test results classified as grades ≥II in cTEE were considered positive for IPVD in the HSS group.

Comparison between cTEE and cTTE for IPVD diagnosis is described in Table 2. At cTEE, 13 patients (68%) with HSS had a positive test for IPVD, one with grade III (moderate) and 12 with grade II (mild). No patient showed significant contrast enhancement. At cTTE, the appearance of contrast in the left chambers was detected in only four (21%) patients with HSS (p < 0.01 versus cTEE). A negative test for IPVD was found in six patients according to both methods (cTTE and cTEE), with contrast test grade I for five patients at cTEE, which was considered normal.

**Table 2:** Comparison between cTEE and cTTE in the diagnosis intrapulmonary vasclar dilatations on patients with hepatosplenic schistosomiasis

	Negative cTTE for IPVD	Positive cTTE for IPVD	Total
Negative cTEE for IPVD	6 (32%)	0	6 (32%)
Positive cTEE for IPVD	9 (47%)	4 (21%)	13 (68%)
Total	15 (79%)	4 (21%)	19 (100%)

cTTE: contrast-enhanced (microbubbles) transesophageal echocardiography; cTTE: contrast-enhanced (microbubbles) transthoracic echocardiography; IPDV: intrapulmonary vascular dilatations

Distribution of clinical, laboratory and echocardiographic characteristics of patients with HSS by the presence or absence of IPVD is presented in Table 3. Age, time of treatment, body surface, and clinical and laboratory data were not different between the patients with and without IPVD. Although A-aO_2_ was not different between the groups, a tendency for higher PaO_2_ values, with significantly higher SatO_2_ and significantly lower PaCO_2_ in the subgroup of patients with and without IPVD.

**Table 3:** Clinical, laboratory and echocardiographic characteristics in patients with hepatosplenic schistosomiasis by the presence or absence of intrapulmonary vascular dilatations at contrast transesophageal echocardiography

	With IPVD (n = 13)	Without IPVD (n = 6)	p value
Age (years)	50.7 ± 8.9	54.7 ± 7.8	0.19
Sex (female/male)	8 F. 5 M	1 F. 5 M	0.14
Time of treatment (year)	15 ± 11	12.5 ± 12	0.30
Body surface index (m^2^)	1.73 ± 0.15	1.70 ± 0.13	0.34
Heart rate (bpm)	70.8 ± 16	79.25 ± 15	0.21
SBP (mmHg)	143 ± 15	131.4 ± 28	0.16
DBP (mmHg)	85 ± 9	77.4 ± 25	0.18
AST (U/L)	34.1 ± 11	35.8 ± 9	0.38
ALT (U/L)	28.2 ± 8	38 ± 21	0.10
Alkaline phosphatase (U/L)	92 ± 27	100.3 ± 25	0.27
Prothrombin time (s)	1.16 ± 0.14	1.04 ± 0.15	0.09
INR	1.25 ± 0.16	1.22 ± 0.3	0.43
Albumin (g/dl)	4.4 ± 0.46	4.5 ± 0.27	0.30
Hemoglobin (g/dl)	13.8 ± 2.4	13.7 ± 2.7	0.48
Hematocrit (%)	40.9 ± 5.2	41.5 ± 6.8	0.42
PaO_2_ (mmHg)	82.5 ± 9.2	75.3 ± 8.9	0.06
PaCO_2_ (mmHg)	34.9 ± 3.5	40.7 ± 5.3	< 0.01
SatO_2_ (%)	96.4 ± 1.2	94.7 ± 2	0.01
A-aO_2_ mmHg	11.0 ± 6.6	10.9 ± 9.84	0.49
PaCO_2_/R mmHg	43.7 ± 4.3	50.8 ± 6.6	< 0.01
Left atrium (mm)	36 ± 5	33 ± 3	0.12
LAV index (mL/m^2^)	24 ± 5	21 ± 3	0.12
LAV (ml)	41.8 ± 9.8	35.9 ± 5.4	0.09
LVDD (mm)	48.5 ± 5.1	45.5 ± 2.9	0.10
LVEF (%)	66,4 ± 0,4	68,7 ± 0,4	0,15
PSP (mmHg)	28,1 ± 4,3	27 ± 3,6	0,35

IPVD: intrapulmonary vascular dilatations; SBP: systolic blood pressure; DBP: diastolic blood pressure; AST: aspartate transaminase (U/L); ALT: alanine transaminase (U/L); INR: international normalized ratio; PaO_2_: partial oxygen pressure; PaCO_2_: partial pressure of carbon dioxide; SatO_2_: blood oxygen saturation; A-aO_2_: alveolar-arterial gradient. PaCO_2_/R: partial pressure of carbon dioxide/respiratory quotient; LAV: left atrial volume; LVDD: left ventricular diastolic diameter; LVEF: left ventricular ejection fraction; PSP: pulmonary systolic pressure.

Considering echocardiographic results, there was a tendency for greater size of the left chambers and for greater left atrial volumes in the subgroup of patients with IPVD, compared with those without IPVD. No significant differences in left ventricular ejection fraction or pulmonary systolic pressure were found between the two subgroups.

The diagnosis of HPS was confirmed in one (5%) patient according to the transthoracic echocardiographic criteria, and in five patients (including the one diagnosed by cTTE; 26%, p = 0.09).

## Discussion

This is the first study to use cTEE for diagnosis of IPVD in HSS. Previous studies have reported the use of cTEE and cTTE for detection of IPVD in cirrhotic patients.^2,8^ Comparison between these two methods for detection of IPVD in cirrhotic patients with portal hypertension was first reported by Vedrinne et al.^10^ According to these authors, cTEE showed higher sensitivity in detecting intrapulmonary shunt compared with the cTTE and is thereby indicated for all patients with suspected IPVD that was not detected by cTTE. In a similar study, Fischer et al.^11^ also found that cTEE was able to quantify and classify the appearance of microbubbles in the left chambers in different grades (minimal to significant), and observed a direct relationship between the level of contrast appearance and pulmonary gas exchange abnormalities.^11^ These studies showed that the use of cTEE was advantageous, since the quality of transthoracic images may make it difficult to visualize the contrast with microbubbles, which is compensated by the transesophageal window.

The presence of intracardiac shunt, originated from a PFO, can be also well identified by cTEE.^9,19,20^ PFO was present in three of the patients excluded from our series after the cTEE and may have been the cause of the passage of contrast to the left chambers and of eventual overestimation of the frequency of IPVD. This shows the superiority of the transesophageal method in the diagnosis of this condition.

The main finding of the present study on HSS patients and relatively preserved liver function was the occurrence of subclinical IPVD and unaltered gas exchange, detected by cTEE in a significantly higher frequency compared with cTTE. In our HSS patients, cTEE was able to detect slight pulmonary vascular changes compatible with initial stages of the disease. The presence of IPVD in HSS had been previously described in our setting by Ferreira et al.^4^ in similar proportion to that observed in our series, but using the transthoracic method only. In our study, the cTEE enabled the detection of IPVD in additional nine patients that had not been diagnosed with the disease by cTTE, which highlights the role of the transesophageal technique in the indirect characterization of this vascular alteration. Therefore, in agreement with previous findings in cirrhotic patients, cTEE in HSS patients was more sensitive as compared with the cTTE for the diagnosis of IPVD, corroborating its superiority in this sense in HSS.

In cirrhotic patients with portal hypertension, the prevalence of IPVD was considerably higher according to cTEE than cTTE, 51-75% and 13-47%, respectively.^10-12,21^ In our study, some of the individuals of the control group showed a mild (grade I) contrast enhancement, probably due to a physiological pulmonary shunt, which facilitates the passage of small contrast microbubbles, capable of passing the pulmonary capillary barrier and reach the chambers. For this reason, grades ≥ II were considered positive for IPVD.

In the present series, there was a tendency of the cTEE to detect HPS in a greater number of subjects compared with cTTE. This can be explained by the diagnostic criteria for HPS, which may vary according to gas exchange parameters.^14^ Maybe the inclusion of a greater number of participants would have led to more significant results.

No statistically significant difference was found in A-aO_2_ between patients with and without IPVD, which highlights the role of cTEE in detecting subclinical disease. On the other hand, three patients with hypoxemia or increased A-aO_2_ showed negative microbubble test by both echocardiographic tests. This may result from the type of contrast (saline solution), the injection technique or the size of the microbubbles injected. Although the microbubble size varied from 24 to180 µ, bubbles greater than 60 µ are more echogenic and possibly more difficult to pass through the capillary bed, even when it is dilated, resulting in a weaker signal.^12^ In addition, the dissociation between the echocardiographic findings and results of the arterial blood gas analysis with respect to IPVD detection reflects the lability of gas exchange; the tests were not performed simultaneously, and hence temporal and dynamic changes of echocardiographic and gas exchange parameters may have occurred. Differences in PaO_2_, SatO_2_, PaCO_2_ between the subgroups of patients with and without IPVD remain uncertain.

Recent studies have suggested that IPVD are precursor or associated diseases of portopulmonary hypertension in patients with cirrhosis and schistosomiasis,^22,23^ and that may be associated with decreased survival.^22^ In cirrhotics, the presence of IPVD worsens the prognosis of portopulmonary hypertension, contributes to an earlier indication of liver transplantation, and increases the risk of mortality after transplantation in patients with increased hypoxemia.^24,25^ However, the prognosis of IPVD in HSS is unknown, and IPVD are present in patients with no clinical or respiratory changes. Further studies are needed to establish the course of this disease.

### Safety

Similar to other studies on patients with cirrhosis and portal hypertension,^11,25^ cTEE was performed without complications in all HSS patients. The probe was safely inserted until the distal third of the esophagus and through the esophageal hiatus for acquisition of transgastric views even in the presence of recent bleeding after sclerotherapy.

### Limitations

One limitation of the present study was the relatively small sample size. However, this is compatible with the profile of the disease in Brazil, whose incidence has been decreasing over the last years. This has been particularly evident in our university hospital which is located away from the endemic zones of the disease.

The low prevalence of HSS in our small sample of patients made it difficult to demonstrate an association of the IPVD with the severity of hypoxemia and increase in A-aO_2_. Another limitation was the fact that the manual agitation of the saline solution produced microbubbles of different sizes, making it impossible to standardize their echogenicity, which would allow higher uniformity of the results.

### Clinical implications

Our findings showed that a negative cTTE does not exclude the presence of cTEE. Therefore, in case of clinical suspicion, cTEE would be indicated to confirm the diagnosis of IPVD in this study group. The clinical relevance and the prognosis of vascular changes in HSS should be determined in future longitudinal studies.

## Conclusions

In patients with HPS and relatively preserved liver function, cTEE was superior to cTTE in detecting IPVD, especially in its earlier stages. The transesophageal method was safe in these patients, most of them with previous sclerotherapy of esophageal varices and without recent bleeding. Finally, PFO, which was diagnosed in these patients, may be a confounding factor for the diagnosis of IPVD at cTTE.

## References

[1] McFaul RC, Tajik AJ, Mair DD, Danielson GK, Seward JB (1977). Development of pulmonary arteriovenous shunt after superior vena cava-right pulmonary artery (Glenn) anastomosi. Circulation.

[2] Krowka MJ, Cortese DA (1990). Hepatopulmonary syndrome: an evolving perspective in the era of liver transplantation. Hepatology.

[3] Ferreira Rde C, Domingues AL, Markman Filho B, Veras FH, Batista LJ, Albuquerque Filho ES (2009). Hepatopulmonary syndrome in patients with Schistosoma Mansoni periportal fibrosis. Acta Trop.

[4] Queirós ASS, Brandão SCS, Domingues ALC, Macedo LG, Ourem MS, Lopes EP (2014). Intrapulmonary vascular dilatation evaluated by 99m Tc-MAA scintigraphy and its association with portal hypertension in schistosomiasis. PLoS Negl Trop Dis.

[5] Rolla G, Brussino L, Colagrande P, Dutto L, Polizzi S, Scappaticci E (1997). Exaled nitric oxide and oxygenation abnormalities in hepatic cirrhosis. Hepatology.

[6] Sogni P, Moreau R, Gadano A, Lebrec D (1995). The role of nitric oxide in the hyperdinamic circulatory syndrome associated with portal hypertension. J Hepatol.

[7] Abrams GA, Jaffe CC, Hoffer PB, Binder HJ, Fallon MB (1995). Diagnostic utility of contrast echocardiography and lung perfusion scan in patients with hepatopulmonary syndrome. Gastroenterology.

[8] Siostrzonek P, Zangeneh M, Gössinger H, Lang W, Rosenmayr G, Heinz G (1991). Comparison of transesophageal and transthoracic contrast echocardiography for detection of a patent foramen ovale. Am J Cardiol.

[9] Vedrinne JM, Duperret S, Bizollon T, Magnin C, Motin J, Trepo C (1997). Comparison of tranesophageal and transthoracic contrast echococardiography for detections of an intrapulmonary shunt in liver disease. Chest.

[10] Fischer CH, Campos O, Fernandes WB, Kondo M, Souza FL, De Andrade JL (2010). Role of contrast-enhanced transesophageal echocardiography for detection of and scoring intrapulmonary vascular dilatation. Echocardiography.

[11] Aller R, Moya JL, Moreira V, García-Lledo A, Sanromán AL, Paino C (1999). Diagnosis and grading of intrapulmonary vascular dilatation in cirrhotic patients with contrast transesophageal echocardiography. J Hepatol.

[12] McLaughlin VV, Archer SL, Badesch DB, Barst RJ, Farber HW, Lindner JR (2009). ACCF/AHA 2009 expert consensus document on pulmonary hypertension: a report of the American College of Cardiology Foundation Task Force on Expert Consensus Documents and the American Heart Association developed in collaboration with the American College of Chest Physicians: American Thoracic Society, Inc.: and the Pulmonary Hypertension Association. J Am Coll Cardiol.

[13] Rodríguez-Roisin R, Krowka MJ, Hervé P (2004). Commitee ETFP-HVDPS: pulmonary-hepatic vascular disorders (PHD). Eur Respir J.

[1] Yock PG, Popp RL (1984). Noninvasive estimation of right ventricular systolic pressure by Doppler ultrasound in patients with tricuspid regurgitation. Circulation.

[15] Currie PJ, Seward JB, Chan KL, Fyfe DA, Hagler DJ, Mair DD (1985). Continuous wave Doppler determination of right ventricular pressure: a simultaneous Doppler-catheterization study in 127 patients. J Am Coll Cardiol.

[16] Fisher MR, Forfia PR, Chamera E, Housten-Harris T, Champion HC, Girgis RE (2009). Accuracy of Doppler echocardiography in the hemodynamic assessment of pulmonary hypertension. Am J Respir Crit Care Med.

[17] Seward JB, Khandheria BK, Oh JK, Abel MD, Hughes RW Jr, Edwards WD (1988). Transesophageal echocardiography: technique, anatomic correlations, implementation, and clinical applicattions. Mayo Clin Proc.

[18] Lewis RR, Hussain A, Rashed KA, Cooke RA, McNabb WR, Chambers J (2001). Patent foramen ovale in elderly stroke patients. Int J Clin Pract.

[19] Homma S, Sacco RL, Di Tullio MR (2002). Effect of medical treatment in stroke patients with patent foramen ovale: patent foramen ovale in Cryptogenic Stroke Study. Circulation.

[20] Pavarino PR, Corbucci HA, de Marchi CH, da Mata PF, de Godoy MF (2004). Contrast echocardiography in the diagnosis of intrapulmonary vascular dilations in candidates for liver transplantation. Arq Bras Cardiol.

[21] Fussner LA, Iyer VN, Cartin-Ceba R, Lin G, Watt KD, Krowka MJ (2015). Intrapulmonary vascular dilatations are common in portopulmonary hypertension and may be associated with decreased survival. Liver Transpl.

[22] Fussner LA, Iyer VN, Cartin-Ceba R, Lin G, Watt KD, Krowka MJ (2015). Carta ao editor: intrapulmonary vascular dilatations are common in portopulmonary hypertension and may be associated with decreased survival. Liver Transpl.

[23] Krowka MJ (2005). Syndrome and portopulmonary hypertension: implications for liver transplantation. Clin Chest Med.

[24] Fallon MB, Krowka MJ, Brown RS, Trotter JF, Zacks S, Roberts KE (2008). Impact of hepatopulmonary syndrome on quality of life survival in liver transplant candidates. Gastroenterology.

[25] Spier BJ, Larue SJ, Teelin TC, Leff JA, Swize LR, Borkan SH (2009). Review of complications in a series of patients with known gastroesophageal varices undergoing tranesophageal echocardiography. J Am Soc Echocardiogr.

